# Deep learning for text summarization using NLP for automated news digest

**DOI:** 10.1038/s41598-025-20224-1

**Published:** 2025-10-17

**Authors:** K. M. Rani Krishna, K. Somasundaram, P. Arulmozhivarman, Sarah A. Immanuel, E. R. Rajkumar

**Affiliations:** 1 Department of Mathematics, Amrita School of Physical Science, Amrita Vishwa Vidyapeetham, Coimbatore, India; 2 Center for Clean Environment, Vellore Institute of Technology, Vellore, Tamilnadu India; 3 School of Analytics and Business Information Systems, Torrens University, Adelaide, Australia; 4 Amrita School of Engineering, Amrita Vishwa Vidyapeetham, Coimbatore, India

**Keywords:** Text summarization, Deep learning, Abstractive text-summarization, EDA, Model evaluation, T5-base, T5-large, BART CNN-large, PEGASUS-large, Computer science, Information technology, Scientific data

## Abstract

Text Summarization, a vital aspect of natural language processing, aims to condense text while retaining its essential meaning. This process is achieved through extractive and abstractive methods. Deep Learning faces challenges in this domain, including semantic understanding, preservation of meaning, efficient handling of long documents, and ensuring coherence and grammatical correctness. Despite these challenges, deep learning offers advantages such as time saving, facilitating information retrieval, scalability, and content personalization. Also, deep learning faces the risk of losing important details, subjectivity in information selection, difficulty in handling complex texts, and variability in summary quality. Addressing these challenges remains an ongoing focus of research and development in the field of NLP. This paper proposes text summarization approach utilizing deep learning models, namely T5-base, T5-large, BART CNN, and PEGASUS. The methodology involves initial data cleaning and preprocessing of the dataset, followed by exploratory data analysis (EDA) to gain insights into the data. Subsequently, the Rouge and BLUE scores of each model are calculated to assess their summarization performance. After training the models, the Rouge and BLUE scores are re-evaluated to measure their effectiveness in generating summaries. The primary objective is to compare the performance of these models based on their Rouge scores, aiming to identify the model that provides the highest Rouge score, indicative of better summary quality. This study contributes to the advancement of text summarization techniques and provide insights into the effectiveness of various deep learning models in this domain.

## Introduction

Considering how people are flooded with an enormous quantity of papers and information due to the internet’s exponential expansion and big data, researchers have been spurred to develop technological solutions to address this challenge. The overwhelming volume of data available online has led to a desire for automated text summarization methods to distil and condense this information into more manageable and digestible forms. Processing lengthy texts consumes significant time and resources, which may not always be feasible or practical.

### Text summarization

**Fig. 1:**
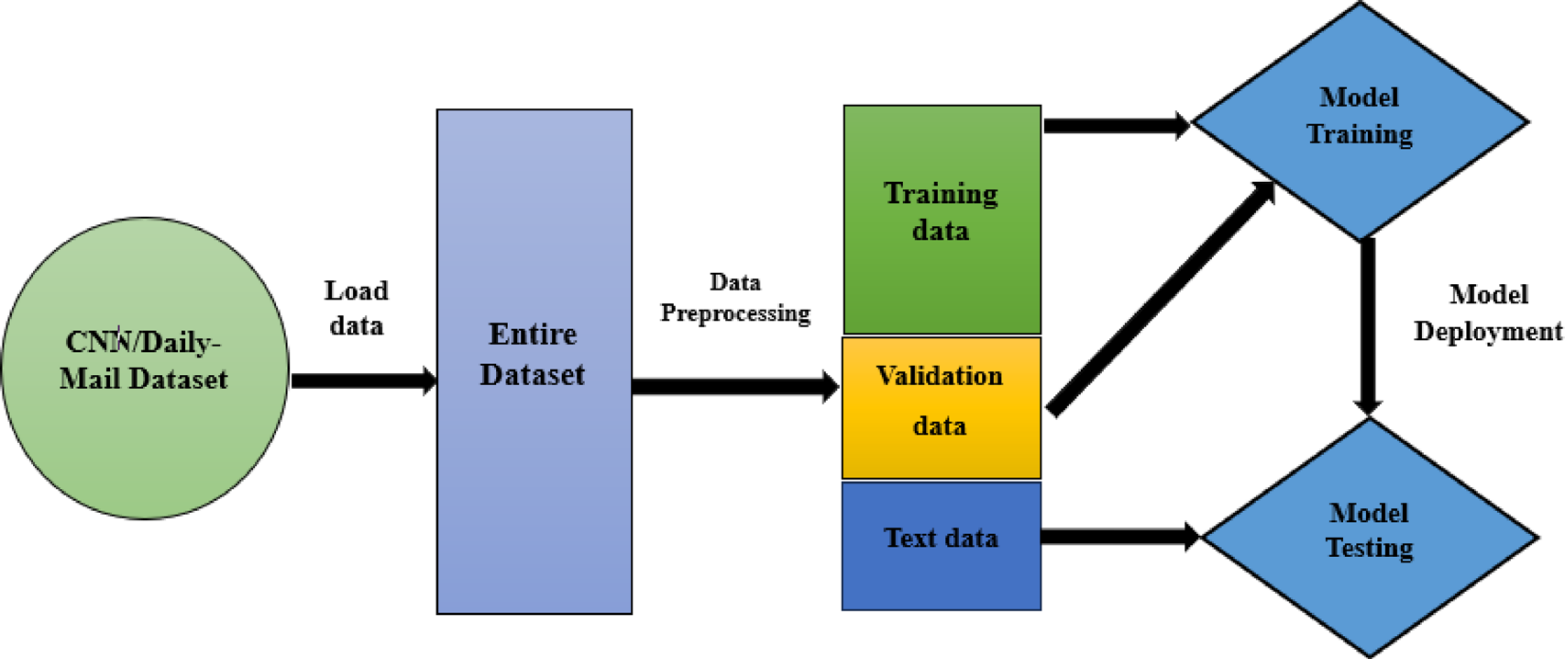
Generating a summary from the input document.

A natural language processing (NLP) technique called text summarizing seeks to summarize a text while keeping its main ideas and important details. Making a condensed version of the original text that highlights the key concepts, ideas, and information is the aim of this. The task of text summary is displayed in Fig. 1. The two primary methods are below:

- *Extractive summarization* Here, the summary is formed by choosing certain sentences or phrases straight from the source text. These selected sentences are typically the ones that contain the most important information, based on criteria such as relevance, importance, and frequency of occurrence.
- *Abstractive summarization* This involves generating a summary that may contain new words or sentences that are absent from the source material. This method seeks to comprehend the text’s content and reword it briefly, potentially using natural language generation techniques.

Text summarization automatically generates a summary from the source document that includes all pertinent information and key phrases. In the same way that people are being overloaded with documents and information due to the internet’s exponential expansion and big data, academics are being pushed to create technical solutions to deal with this issue. The over whelming volume of data that is available online has led to a desire for automated text summaries to distil and condense this information into more manageable and digestible forms.

With the proliferation of digital content, text summarization has become crucial for managing the overwhelming amount of information, particularly in the context of news articles. Modern news dissemination involves various modes, such as text, images, videos, and audio. While the textual content is essential, the context provided by accompanying media significantly enhances understanding. This complexity necessitates advanced summarization techniques that go beyond extracting key sentences to consider the broader context and multimodal inputs.

**Fig. 2:**
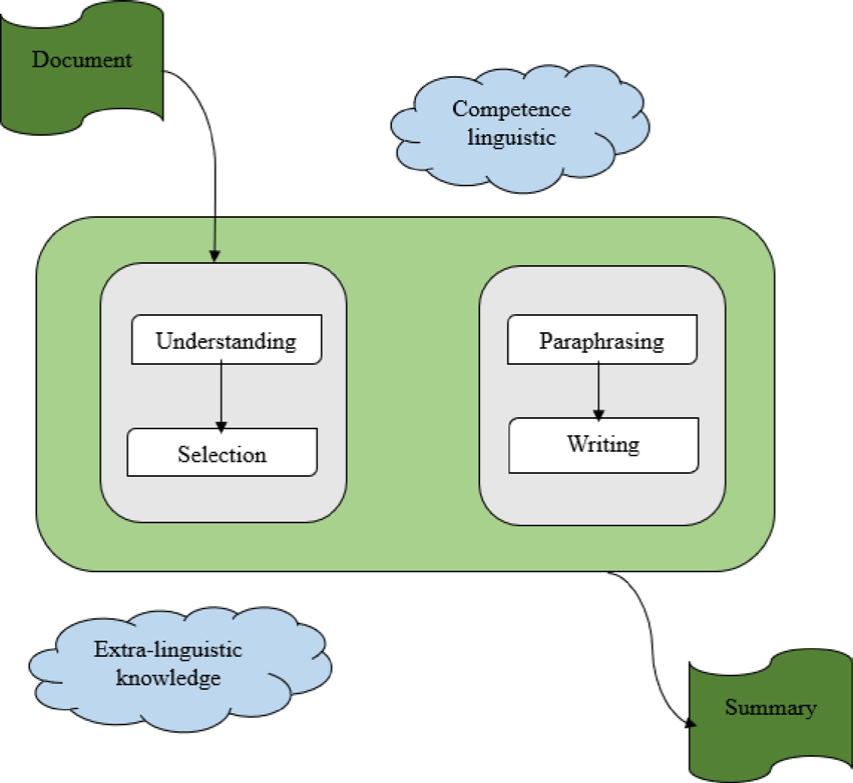
Humans’ process for generating summaries.

Understanding a summary through human resources involves reading the text thoroughly, identifying key points and main ideas, and condensing the content into a shorter form. Figure 2 shows how humans produce summaries of a given original text document. This includes understanding the context, recognising significant details, and rewording the data in a way that makes sense and is clear.

Here is how text summarization addresses these demerits:

- *Information overload management* Long texts often contain redundant or irrelevant information, making it challenging for readers to identify the most pertinent points. Text summarization condenses lengthy texts into concise summaries, highlighting key information and reducing the cognitive load on readers.
- *Time efficiency* Reading lengthy texts can be time-consuming, especially when users need to extract specific information quickly. Text summarization facilitates rapid information retrieval by generating succinct summaries that capture the main point of the text. This time-saving aspect is particularly valuable in scenarios where time is limited, such as during research or decision-making processes.
- *Resource optimisation* Processing and analysing very long texts require significant computational resources and human effort. It helps optimise resource utilisation by reducing the volume of text that needs to be processed. Instead of analysing the entire document, users can focus on the summarised version, leading to more efficient resource allocation and improved productivity.
- *Enhanced accessibility* Long texts can be daunting for individuals with limited time, attention span, or cognitive abilities. A summarised version can be particularly beneficial for students, professionals, and individuals with diverse learning needs, enabling them to access and understand information more effectively.

In response to this, researchers have turned to technological approaches, such as NLP, machine-learning, and deep learning, to automatically summarize text. These approaches leverage computational algorithms to analyse and extract key information from textual data, generating brief synopses that highlight a document’s key elements.

This work seeks to improve upon existing text summarization methods, ensuring better handling of linguistic complexities and providing more precise, contextually aware summaries.

### Literature review

The study^1^ primarily focuses on Arabic abstractive text-summarization using deep learning techniques. For Arabic summarization, this study compares and utilises the performance of several model architectures, such as Transformer and RNN based models, as well as pre-trained language models like AraBERT, AraGPT2, and AraT5. Another deep learning method for abstractive text summarization in Arabic is the study by Khalil et al.^2^. To create logical and contextually appropriate summaries, the authors examine the difficulties and developments in producing summaries that go beyond simply extracting sentences. The project advances natural language processing (NLP) by tackling the unique linguistic challenges associated with summarising Arabic texts.

The study^3^ discusses Automatic Text summarization (ATS) and highlights the need for it, given the explosion of internet text material. To enhance the quality of summaries, the authors proposed placing greater focus on abstractive and hybrid techniques. The work of Rahul and Monika^4^ provides an overview of machine learning techniques used in the NLP field for text summarization. They cover a range of techniques and their uses, giving readers a basis for comprehending machine learning’s function in textual data summarization.

The importance of Automatic Text Summarization (ATS)^5^ is growing due to the abundance of internet content. ATS helps users efficiently retrieve important details by condensing information into brief summaries. Researchers have been working on improving summarization strategies with machine learning and optimisation techniques. This work also discusses abstractive and multilingual summarising. It goes over both intrinsic and extrinsic assessment techniques and identifies areas for future studies that improve the effectiveness of ATS. Pan et al.^6^ proposes a sequence-to-sequence text summarising model with a topic-based attention mechanism by emphasising topic-relevant information, this methodology improves the summary process and can provide more accurate and contextually aware summaries. This work integrates scene influence through word topic distribution to overcome a shortcoming in automatic summarization. Implicit information is not taken into consideration in traditional Seq2Seq models.

Gupta et al.^7^ studied the automated news summarization using Transformer based pre-trained models. It addresses the need for efficient summarization due to the vast amount of online text data. Results indicate the T5 model excels, suggesting potential for future work in enhancing model robustness. Similarly, in the research paper^8^, Transformer-based models (GPT, T5, BART, and PEGASUS) for news article summarization are compared. In that it is discovered that T5 performs better in ROUGE metrics because of efficient fine-tuning.

The importance of text summary in handling the information overload brought on by internet technology is covered in this paper^9^. The need for automatic text summarization in handling the growing number of scientific publications is discussed in the paper^10^ which compares the high accuracy of LSTM and GRU algorithms. Analyses using ROUGE indicators show that the Latent Semantic Analysis (LSA) approach and its effectiveness are similar. Also, Rahman and Siddiqui^11,12^ proposed convolutional LSTM for abstractive text summarization.

PEGASUS, a unique pre-training approach for abstractive text summarization based on extracted gap-sentences, was introduced by Zhang et al.^13^. By filling in the blanks in a text, this method enables the model to comprehend context more fully and produce summaries that are more accurate. This research sheds light on deep learning models’ pre-training stages for summarization tasks. The use of pre-training in natural language production for text summary is covered in this study by Zhang et al.^14^. They investigate how pre-training can enhance generated text quality, resulting in more coherent and fluid writing. The study is important for comprehending how summarization model performance is affected by the pretraining phase.

The framework proposed in^15^ integrates knowledge and topic extractors to improve accuracy and control. This is the conference version of the previously mentioned PEGASUS paper, in which Zhang et al.^16^ provides a more formal presentation of their results. The performance of the PEGASUS model and its implications for the field of NLP are discussed in detail in this publication.

Yujia and Jan^17^ applied the reinforcement learning to abstract text summarization. They have used encoder-decoder model with a hierarchical attention mechanism and multi-objective reinforcement learning for the abstract text summarization. In 2024, Supriyono et al.^18^ wrote a survey article on the text summarization. Also, machine learning and graph-based approaches for text summarization were studied in^19,20^.

### Significance of the research

In recent years, Automatic Text Summarization (ATS) techniques become important in different fields such as scientific reports, news articles, legal document and Internet. In these fields, the textual contents are growing exponentially due to large amount information. Developing an appropriate tool for the ATS is a challenging problem. This paper addresses a deep learning model for the ATS.

#### Contributions

**Fig. 3:**
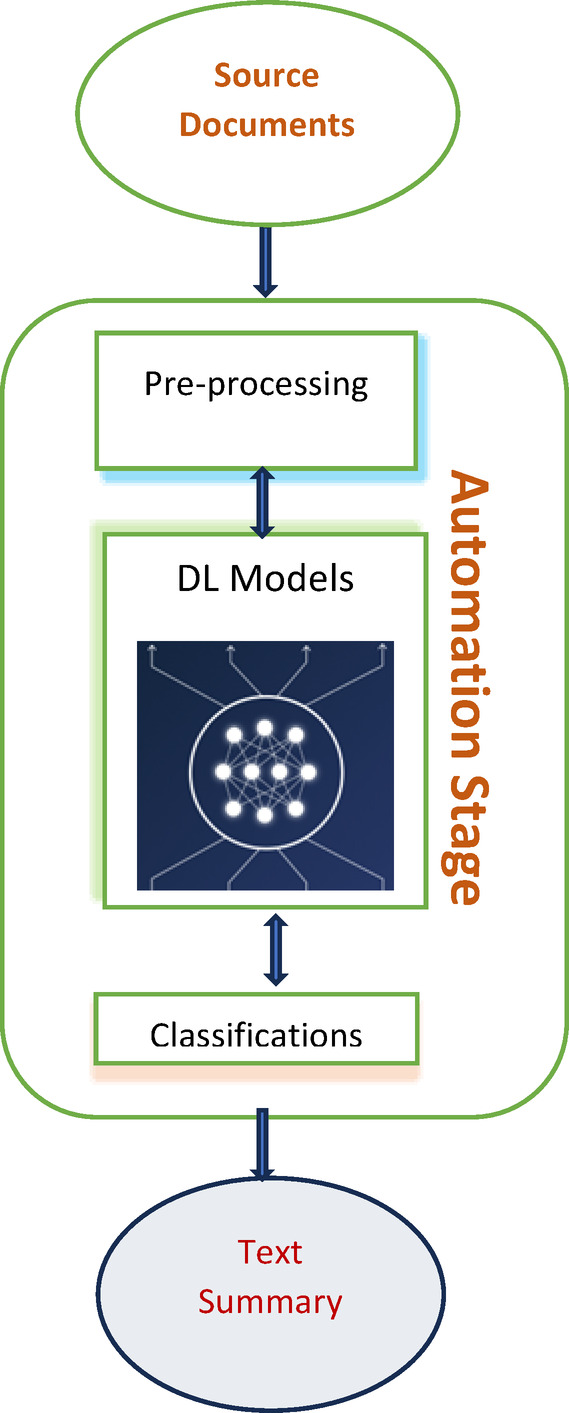
Flow chart.

- We have taken CNN/Daily-Mail dataset. We preprocess the data by using tokenization, normalisation, truncation and attention masks techniques.
- Exploratory data analysis is done to understand the insights of the date.
- We propose deep learning models, namely T5-base, T5-large, BART CNN, and PEGASUS for the text summarization.
- Rouge scores and BLUE scores of each model are calculated to assess their summarization performance. After training the models, the Rouge and BLUE scores are re-evaluated to measure their effectiveness in generating summaries.
- We analyze the models based on different paraments such as Rouge scores and Blue scores.
- Our experimental results show that BART CNN model gives better performance than the other deep learning models.
- The entire process is given as a flow chart in Fig. 3

## Data pre-processing

### Structure of the dataset

The dataset is typically divided into three main CSV files:

- train.csv

There are approximately 287,113 articles and summaries. This split is used to train the summarization model, and it contains a bulk of data, which allows the model to learn the mapping from articles to summaries.

- validation.csv

There are approximately 13,368 articles and summaries. This split is used to validate the model during training. It helps in tuning hyperparameters and provides an unbiased evaluation to prevent overfitting. The performance tells us how well the model performs.

- test.csv

There are approximately 11,490 articles and summaries. This is used to evaluate the final performance of the trained model. It serves as the ultimate test to assess the model’s generalisation capability.

A separate split of the dataset used for training, validation, and testing, respectively. The data set is contained in each csv file. By creating these divisions, overfitting is prevented, and generalisation performance is evaluated by allowing models to be trained, adjusted, and assessed on various subsets of the data.

The dataset comprises three main columns:

- *id* A unique identifier for each article-summary pair.
- *article* The full text of the news articles from CNN and the Daily Mail.
- *highlights* The human-written summaries of the articles, capturing the essence of the full text.

Our aim is to support the building the models that shorten lengthy text paragraphs into one or two sentences. It should be made clear that while highlights generated by models trained on this dataset are automatically generated, they represent the language used in the articles.

**Fig. 4:**
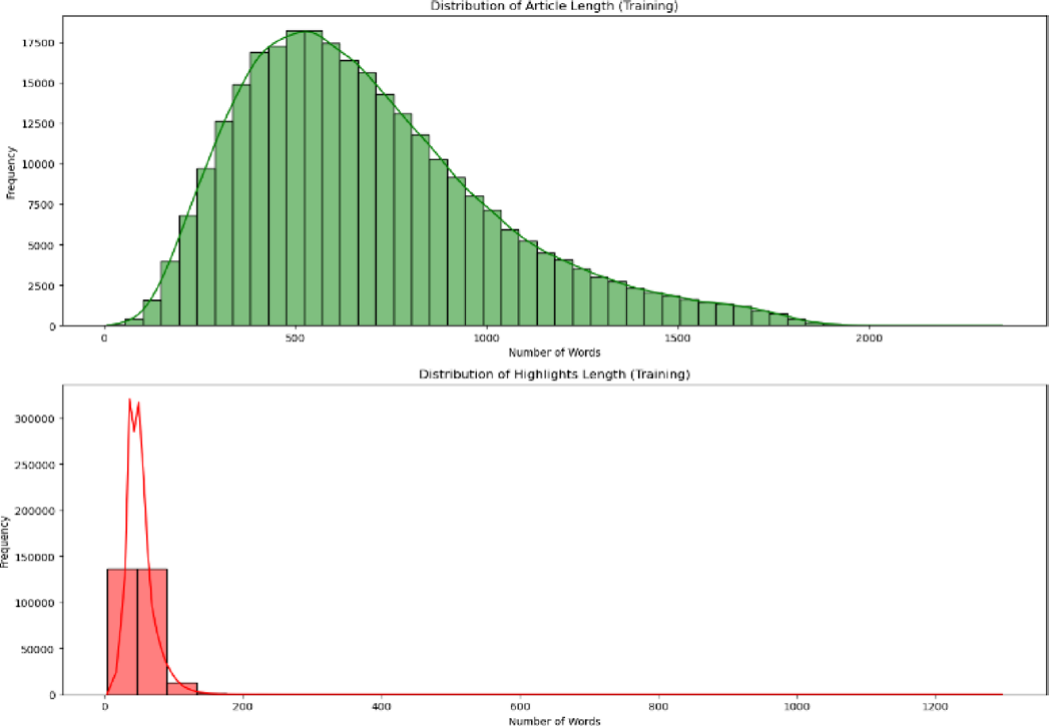
Average count of Article and Highlights Length.

We initially extracted the CNN/ Daily Mail dataset from the website Kaggle. To learn more about the structure of the data, understand the dataset better, and prepare it for training and evaluation, we conducted a data length analysis. We calculated the average length of the articles and highlights for each split of the dataset, as this step is crucial in understanding the distribution of text lengths, which directly impacts the performance and efficiency of summarization models. The average count of articles and highlights length are shown in Table 1 and Fig. 4.

**Table 1:** Average count of Article and Highlights Length.

Sl. no	Splits	Average article length (in words)	Average highlight length (in words)
1	Training	692	52
2	Validation	676	58
3	Testing	683	55

*Data characteristics* Finding average lengths aids in comprehending the basic characteristics of the data. It offers information about the article details and the concision of their highlights.

*Normalisation and scaling* Determining whether the text data must be scaled or normalised for additional processing depends on its average length.

Also, summarization models like transformer-based models often have limitations on the maximum sequence length they can process. So, it is easier to configure the model correctly when we are aware of the average lengths.

Average lengths are helpful to know when evaluating evaluation metrics. For example, in our paper, the length of the text may affect BLEU or ROUGE scores; longer texts may have different metric values than shorter ones. An average highlight length could mean that a summary model must be modified to generate a shorter summary, whereas greater average lengths could indicate the need for summaries that are more in-depth.

*Human review* Implementing these statistics researchers and data scientists will develop a deeper understanding of the dataset and make well-informed choices regarding data preparation, cleaning, and annotations. For human review or marking, lengthy articles, for instance, might need to be divided into smaller sections.

Data preprocessing is a crucial step to prepare textual data for text summarization model training and evaluation. The raw text data is ready for machine learning algorithms after a few steps to clean, normalise, and arrange the data are implemented.

### Key steps in data preprocessing

- *Tokenization* Breaking down text into smaller units called tokens, typically words or subworlds. The purpose of this is to convert raw text data into a structured format that models can capture and understand easily.
- *Normalisation* Converting text data to a standard format, such as lowercase, removing punctuation, and handling special characters.
- *Padding and truncation* Text sequences can be made to a prearranged length by truncating lengthier sequences or inserting padding characters. The main purpose is to maintain uniform input sizes for effective batch processing and training of models.
- *Creating attention masks* Create binary masks to distinguish between padding tokens and real tokens. It helps the model disregard padding tokens during inference and training. By ignoring padding tokens, attention masks assist the model in concentrating on the pertinent portions of the input sequence. By doing this, the model’s attention processes are guaranteed to function properly, improving its ability to produce accurate summaries.

### Advantages

- *Hardware used* The proposed model was trained and tested on a system with the following configuration: Intel Core i3-8145U CPU @ 2.10 GHz 2.30 GHz, 12.0 GB RAM, running Windows 11 version 23H2.
- *Efficiency and consistency* For batching and parallel computation in deep learning models, this preprocessing makes sure that every input and output sequence has a constant length. The model reduces computing costs by handling inputs of different lengths efficiently without making dynamic improvements by padding and truncating sequences to set lengths.
- *Model performance* Proper tokenization ensures that the input of the model follows expected formats, including padding and truncation. For training steadiness and convergence, this consistency is essential.
- *Reproducibility* To maintain reproducibility, the preprocessing pipeline employs standardised tokenization and padding approaches. It is simpler to reproduce and confirm results when preprocessing techniques have consistent behaviour in the model.
- *Memory management* The function effectively controls memory usage by capping the sequence lengths to 512 tokens for inputs and 150 tokens for outputs. This prevents potential problems with extremely long sequences that might utilise available memory during training.
- Learning rate for the T5-Base is 0.0003 using the optimizer AdaFactor. The preferred scheduler is linear with warmup steps 500–2000. Batch size 32 and input length 512–1024 tokens (maximum source and target lengths) with 5 Epochs.

The hyperparameter details for the four models are shown in the Table 2.

**Table 2:** Hyperparameter details for the four models.

Parameter	T5-base	T5-large	BART	PEGASUS
Model size	220 M	770 M	139 M	568 M
Max input length	512 tokens	512 tokens	1024 tokens	1024 tokens
Max target length	128 tokens	128 tokens	128 tokens	128 tokens
Batch size	64	32	64	64
Learning rate	3e−4	3e−4	3e−5 to 5e−5	1e−4
Optimizer	AdamW	AdamW	Adam	Adam
Adam β1, β2	β1 = 0.9, β2 = 0.999	β1 = 0.9, β2 = 0.999	β1 = 0.9, β2 = 0.999	β1 = 0.9, β2 = 0.999
Weight decay	0.01	0.01	0.01	0.01
Dropout	0.1	0.1	0.1	0.1
Learning rate schedule	Linear with warm-up	Linear with warm-up	Linear decay with warm-up	Linear decay with warm-up
Warm-up steps	2000	2000	2000	2000
Training steps	100–200 K	50–100 K	30–60 K	30–60 K

Text summarization focuses mainly on data preparation, which converts unstructured data into a standardised structure that is helpful in model training. By ensuring effective processing, precise representation, and structural clarity of the input data, it improves model performance. Appropriate preprocessing produces more dependable and efficient summarising models that provide the highest-level summaries. This technique is essential for preparing data for Seq2Seq models since it defines input lengths, increases determining efficiency, and improves model performance and reproducibility.

## Models used

To assess how well four cutting-edge deep learning models performed in producing acceptable and succinct summaries of a news dataset, we used them in this study. The models used in this paper are T5-base, T5-large, BART-CNN-large, and PEGASUS-large. Prior to delving into the detailed analysis of these models, we discuss the evaluation metrics used to identify the most effective model for text summarization. Specifically, we used ROUGE and BLEU scores as our primary metrics for performance assessment. These metrics and how we used them in our study are further examined in the sections below.

### ROUGE score

ROUGE (Recall-Oriented Understudy for Gisting Evaluation) scores are essential for assessing how effectively the text summarization algorithm works. These metrics serve as benchmarks for the quality of summarization algorithms by objectively measuring the degree to which machine-generated summaries align with human-written summaries. It ranges from 0 to 1, and as the value of the rouge score is closer to 1, the model is more perfect and accurate. In the field of NLP and summarization, ROUGE scores are used to validate the efficacy of proposed methods, ensuring transparency and reproducibility of research findings.

In this paper, we used three rouge score metrics as follows:

1. *ROUGE-1 (Unigram Overlap)* The overlap of unigrams, or single words, between the generated summary is measured by ROUGE-1. The F1-score, accuracy, and recall are computed by counting the number of overlapping unigrams.
  $${\text{Precision }} = \frac{{{ }Number\;of\;overlapping\;unigrams}}{Number\;of\;unigrams\; in\;generated\;summary}$$
  $${\text{Recall}}\;{ = }\;\frac{{{ }Number\; of\;overlapping\;unigrams}}{Number\;of\; unigrams\; in\;reference\;summary }$$
2. *ROUGE-2 (bigram overlap)* The overlap of bigrams, or pairs of adjacent words, between the reference summary and the generated summary is measured using ROUGE-2. It broadens the assessment to include additional semantic data.
3. *ROUGE-L (longest common subsequence)* The longest common subsequence (LCS) between the reference summary and the generated summary is measured by ROUGE-L. Instead of concentrating on word overlap, it considers the longest word sequence that appears in both summaries.

By evaluating the degree to which generated summaries match the summaries authored by humans, ROUGE scores shed insight into the quality of these summaries. Better summarising performance is demonstrated by higher ROUGE scores, and every measure presents a unique angle on the summarization procedure. ROUGE-2 builds on ROUGE-1’s surface-level lexical overlap recognition to include bigrams and more contextual information. When analysing the longest common sequence, ROUGE-L places a strong emphasis on content overlap, which is essential for accurately summarising the original text. All things considered, these requirements are essential for directing the creation and assessment of text summarising systems since they offer an objective assessment of summary quality.

### Bleu score

Text summarization and machine translation systems are evaluated on their text quality using standards called BLEU (Bilingual Evaluation Understudy) scores. They evaluate the degree to which the generated text is comparable to reference texts, which are usually summaries or translations provided by humans. Although BLEU is more frequently linked to machine translation, text summarization also makes use of it. The scores range from 0 to 1, where 1 indicates a perfect match between the generated summary and the reference summary. However, achieving a score close to 1 is very rare.

Role in Text Summarization:

- *Precision-based metric* When contrasting the reference summaries with the generated summary, BLEU analyses the accuracy of n-grammes, or sequences of n-words. This indicates that the number of n-grammes in the generated text matches those in the reference text.
- *Multi-ngram analysis* BLEU preserves both surface-level vocabulary matches and more complex contextual matches by considering unigrams, bigrams, trigrams, and higher-order n-grams. This offers an accurate assessment of the substance and flow of the summary.

BLEU scores offer an accepted technique for comparing and enhancing the field by offering a standard for evaluating the efficacy of various summarization methods.

$${\text{BLEU }} = {\text{ BP }} \times {\text{ exp}}\left( {\mathop \sum \limits_{n = 1}^{N} \omega_{n} \log p_{n} } \right).$$

### T5 base

Text summarization is greatly impacted by the T5 (Text-to-Text Transfer Transformer) model, particularly in its T5 base version, because of its robust and flexible architecture. Google Research created T5, which standardises NLP tasks into a text-to-text format, making a variety of linguistic operations, including summarization, more efficient. Modelling hidden text lengths enhances contextual and semantic understanding during pre-training. The models’ ability to produce clear and coherent summaries are optimised through post-training fine tuning on certain summarising datasets.

The role of it in text summarization lies in handling long inputs, generating summaries, customization and versatility. By recognising connections and relationships within the text, the transformer architecture of T5-base effectively handles extended text sequences, which are essential for summarising jobs. It is also able to generate summaries that accurately capture the major ideas of the original text due to its encoder-decoder architecture, which has been improved by intensive pre-training. It is also flexible enough to be used in a variety of areas and can be adapted to meet varied summarization demands, such as summarising studies on news items. With its dominant transformer-based development and unified approach, it is essential for text summarization. It is an important tool in NLP given its capacity to handle lengthy texts, produce logical results, and adjust to various jobs.

The initial test performed when testing the dataset using the T5 base model produced a moderate initial result, with an average ROUGE score of approximately 0.29 and a BLEU score of 0.0431. Table 3 shows the average scores during testing the dataset. The score shows a considerable improvement following the model’s fine-tuning using a task-specific dataset. Also, this improvement shows how fine-tuning makes a difference in helping the model accurately capture and recreate important aspects of the source text in its summaries. The outcome shows how well the T5 base model can summarise text, particularly when adjusted to the unique features of the dataset, which produces summaries that are more precise and logical.

**Table 3:** Pre-training for T5-base.

Model	Average scores
Average ROUGE-1	0.29
Average ROUGE-2	0.1071
Average ROUGE-L	0.2015
Average BLEU	0.0431

### T5-LARGE

A member of the transformer family, the T5 large model was developed by Google Research. Every NLP task is considered by the T5 architecture as a text-to-text task, implying that text strings are consistently utilised for both the input and output. This method provides the use of a single framework for a variety of tasks, including text summarization, question answering, and translation. In comparison with smaller models, the T5 large is superior and capable of handling complex language interpretation and creation tasks since it has 24 layers and 770 million parameters. Using an elimination auto-encoding objective, the model is pre-trained on a large corpus of different text data, where it gains the ability to detect missing tokens in a distorted input sequence. This extensive pre-training helps in T5’s development of strong linguistic knowledge.

Its role of it in text summarization, it excels at text summarising, generating clear, understandable summaries of lengthy texts. It is capable of capturing complex connections and interpretations inside the text because of its immense parameter count and thorough pre-training. Since it enables the model to autonomously handle the task as a sequence-to-sequence problem, the content-to-text framework is highly beneficial for summary. It converts an input document (source text) into a brief version (summary), preserving important context and information.

**Fig. 5:**
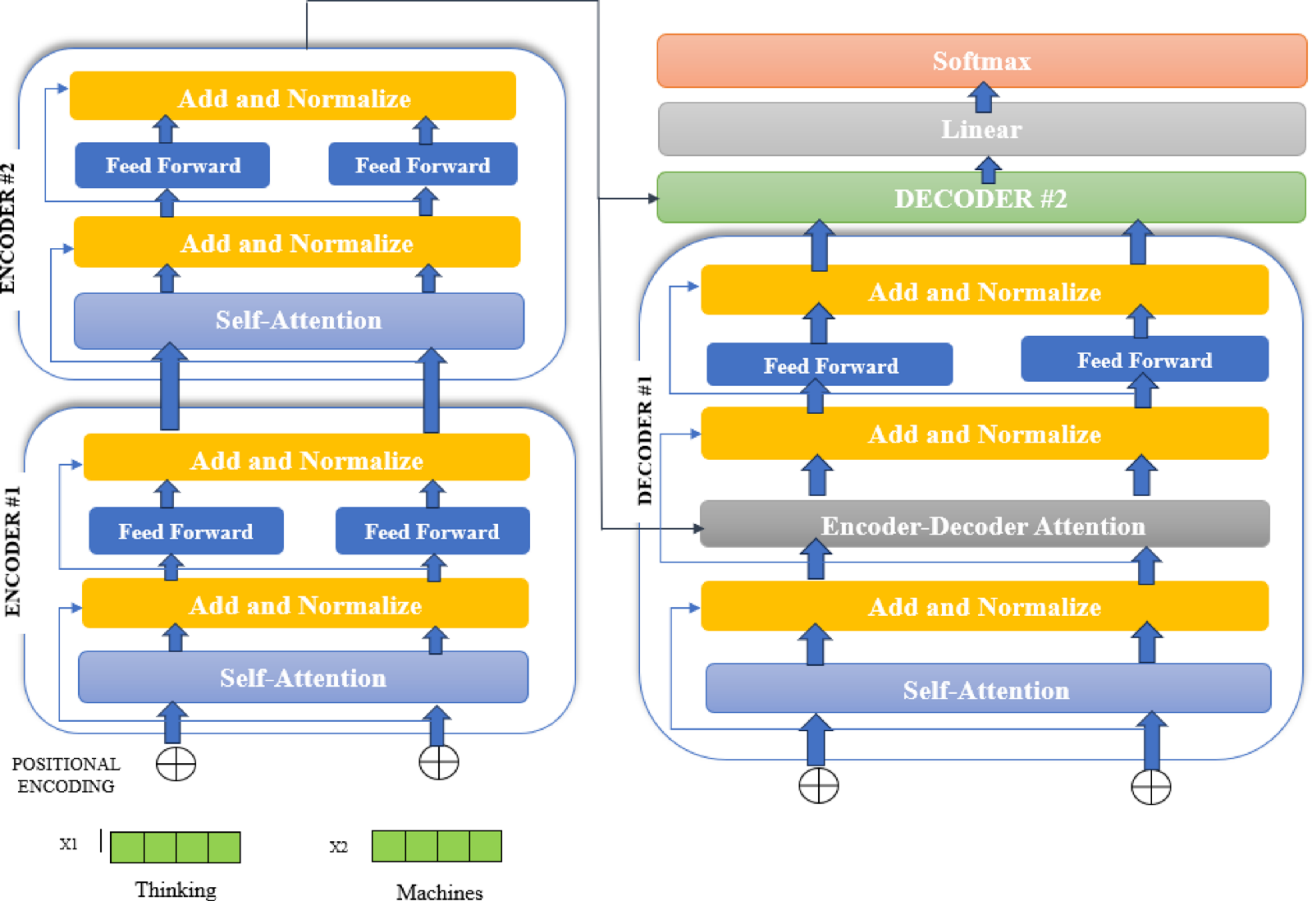
T5- model structure.

To use T5-large for summarization, the pre-trained model needs to be modified using a specific summarization dataset. In the process of fine-tuning, the model develops the capacity to provide summaries that are relevant to the style and requirements of the dataset. T5-large’s strong language processing abilities ensure that the summaries generated are not only grammatically and naturally correct but also informative. It is a useful tool for managing the massive quantity of textual information encountered in modern applications since it can provide high-quality, coherent, and short summaries by utilising its extensive pre-training and smart architecture. The model structure of T5 is shown in Fig. 5.

In the initial testing of the dataset using T5-large, the output average ROUGE score was approximately 0.2429 and the BLEU score was 0.0338. Table 4 shows the average rouge and bleu scores of testing the dataset. As we see, compared to T5-large, T5-base has a higher score.

**Table 4:** Pre-training for T5 large.

Model	Average scores
Average ROUGE-1	0.2429
Average ROUGE-2	0.0829
Average ROUGE-L	0.2682
Average BLEU	0.0338

### BART-LARGE-CNN

Facebook AI developed the transformer-based BART (Bidirectional and Auto-Regressive Transformers) model specifically for sequence-to-sequence tasks like text summarization. A modified version of the BART model known as the “large CNN” variant includes a huge architecture with convolutional neural networks (CNNs) integrated into its layers. The benefits of auto-regressive and bidirectional transformers merge in BART. It consists of a left-to-right auto-regressive decoder, like GPT, that generates output text one token at a time, and a bidirectional encoder, like BERT, that scans the full input text to grasp context. Because of its dual nature, BART can handle jobs that call for producing coherent and contextually relevant text (output sequence) as well as understanding context (input sequence).

In the process, BART-large CNN plays an important role by transforming lengthy and complex text into a shorter summary while retaining the original meaning and key information. It works in two parts: encoding and decoding. In encoding, the bidirectional encoder reads the whole input text and identifies all the complex dependencies and relations that are present in it. If the model fully understands the context is essential for producing accurate summaries. Utilising encoder’s context, the auto-regressive decoder predicts each word individually to provide the summary. The output summary is kept coherent and flexible, which helps with this step-by-step production.

**Fig. 6:**
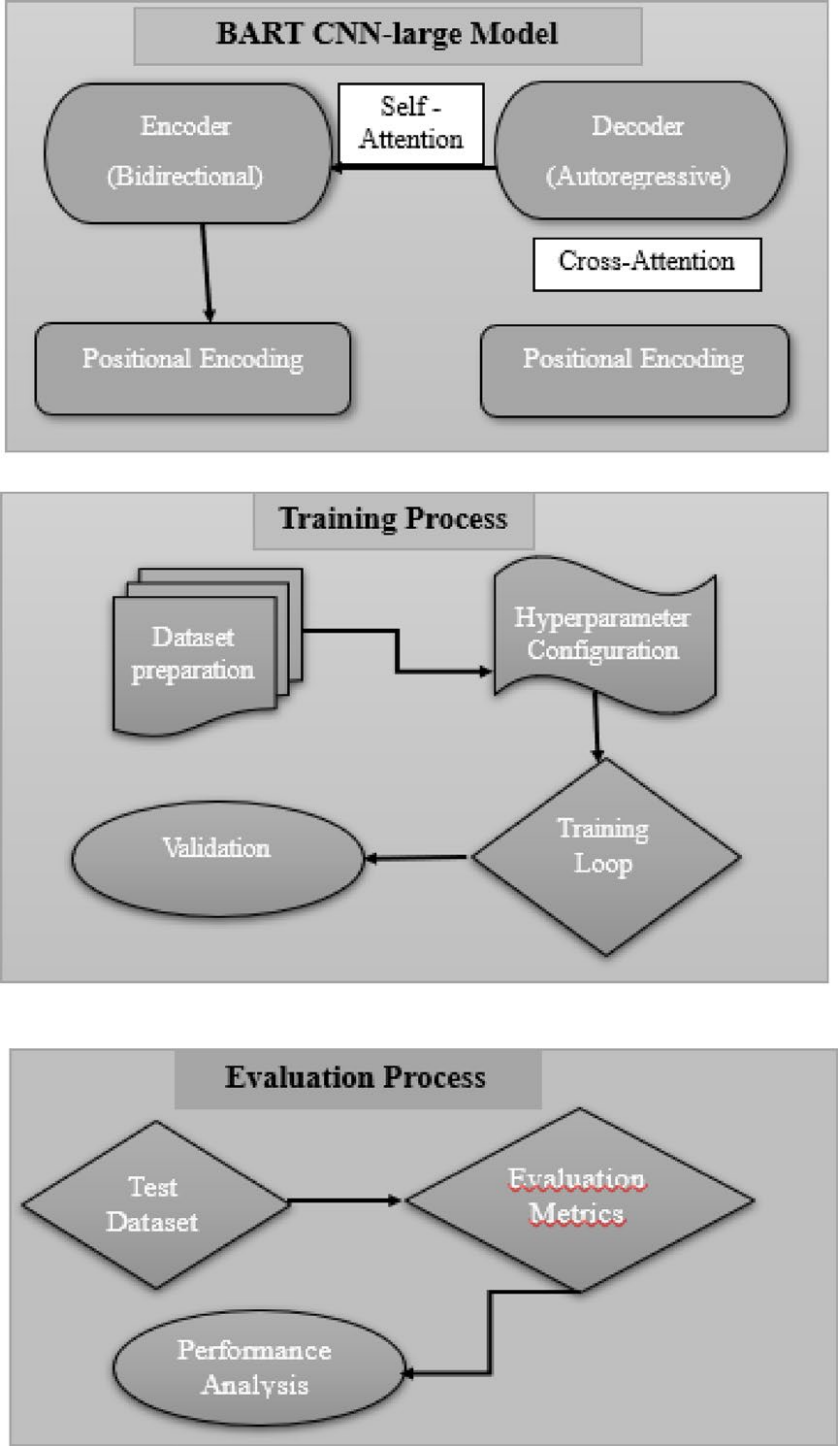
Workflow of BART CNN-large model.

The advantage lies in using a decoding autoencoder technique, BART is pre-trained on a sizable corpus of text, where it gains the ability to reconstruct original text from corrupted versions. This improves its text comprehension and production skills. Its summarising skills are further improved by fine-tuning certain summarization datasets. Overall, it leverages its sophisticated architecture and pre-training to produce text summaries of higher quality, making it an efficient tool in NLP. The workflow of BART CNN-large model is shown in Fig. 6.

When we performed the initial test using BART-large CNN to establish baseline performance metrics, the average ROUGE score and BLEU score were roughly 0.1352 and 0.0182, respectively. The average rouge and bleu scores obtained from testing the dataset are given in Table 5.

**Table 5:** Pre-training for BART-large CNN.

Model	Average scores
Average ROUGE-1	0.1352
Average ROUGE-2	0.0628
Average ROUGE-L	0.0948
Average BLEU	0.0182

### PEGASUS-large

The most advanced model built specifically for text summarising tasks is called PEGASUS (Pre-training with Extracted Gap-Sentences for Abstractive Summarising Sequence-to-Sequence). PEGASUS is a highly efficient method for producing high-quality summaries because it uses a new pre-training objective developed for summary, which was created by researchers at Google Research. The Transformer architecture, a deep learning model built for NLP tasks, constitutes basis for PEGASUS. The encoder-decoder structure of PEGASUS is similar to that of other Transformer models. The input text is analysed by the encoder, and the summary has been generated by the decoder.

PEGASUS’s pre-training methodology is its main innovation. It includes a novel technique known as “Gap-Sentence Generation (GSG),” in contrast to traditional models that have already been pre-trained with generic language modelling tasks. The key terms in GSG are masked off, and the model learns how to recognise these gaps from the rest of the text. By closely aligning the pre-training job with the summary, this methodology improves the model’s capacity to produce concise and understandable summaries. PEGASUS can generate summaries of excellent quality, is flexible enough to work in a variety of fields and minimises the amount of human labour required for text summaries.

When we performed initial testing with Pegasus-large to establish baseline performance metrics, the average ROUGE score and BLEU score obtained were 0.2493 and 0.0317, respectively. Table 6 shows the values corresponding to pre-training for PEGASUS-large and Table 7 shows the baseline performance metrics for various models.

**Table 6:** Pre-Training for PEGASUS-large.

Model	Average scores
Average ROUGE-1	0.2493
Average ROUGE-2	0.0850
Average ROUGE-L	0.1664
Average BLEU	0.0317

**Table 7:** Pre-Training results.

Baseline performance metrics (pre-training)					
Sl. no	Models	ROUGE-1	ROUGE-2	ROUGE-L	BLEU
1	T5-base	0.29	0.1071	0.2015	0.0431
2	T5-large	0.2429	0.0829	0.1682	0.0338
3	Bart-large CNN	0.1352	0.0628	0.0948	0.0628
4	PEGASUS-large	0.2493	0.0850	0.1664	0.0317

## CNN/daily-mail news

The CNN/Daily-Mail dataset is sourced directly from the Hugging Face ‘datasets’ library^20^. A well-known resource for building and evaluating text summarising models is the CNN/Daily Mail dataset. Since its initial introduction in 2015 by Karl Moritz Hermann and associates, it has established itself as a standard for evaluating summarization algorithms’ performance. This dataset is perfect for extractive and abstractive summarization models because it includes summaries of news stories from CNN and the Daily Mail. The dataset contains over 300,000 unique news articles.

## Results and discussions

Using the CNN/Daily Mail dataset, this study attempts to assess how well different deep learning models perform on the text summarising task. The technique comprises multiple pivotal steps: gathering data, preparing data, training and testing models, and assessing performance. T5 base, T5 large, BART CNN large, and PEGASUS large are the models that are assessed in this investigation.

As we discussed already, the paper involves these steps:

*Data collection*: This study uses the CNN/Daily-Mail dataset. The news documents in this dataset, along with the summaries that go with them, are suitable for training and testing text summarization models.

*Data preparation*: Reduces unnecessary characters, HTML tags, and non-text elements to ensure consistency and quality. To ensure stable sequence lengths, text is tokenized into sub- word units, padded, and shortened. In order to help the model stay focused during training, attention masks have been created to distinguish between real tokens and padding.

*Model-Training*: In this study, the model-training process involves multiple organised phases, each of which improves the models’ ability for summarization. Below is an overview of the methods that were used:

- *Setting training parameters* To effectively direct the training process, key hyperparameters like learning rate, batch size, epochs, and methods for optimisation are carefully set. Based on accepted best practices for deep learning model training, these parameters were chosen.
- *Training the models* The models are successively trained to reduce the loss function using gradient descent and backpropagation. Through this procedure, the models can improve their capacity to produce precise summaries by gaining insight from the subtleties of the CNN/Daily-Mail dataset.
- *Validation* To assess how well the models performed and avoid overfitting, they are frequently assessed on the validation set. To make sure the models generate clear summaries and are well-generalised, this required analysing a variety of metrics.
- *Iterative optimisation* Continuous improvements are made to the training procedure based on validation outcomes. The models can gradually enhance their summarising skills because of this iterative process.
- *Generalisation* The models’ strong performance in summarising news items from the dataset is ensured by their rigorous training and validation, which showed that they could generalise well to previously unseen material.

Our four models are effectively taught to produce precise and perceptive summaries by closely adhering to this extensive training process, which offered insightful information about the news items’ content from the dataset.

Performance Evaluation: ROUGE and BLEU scores are used to assess the models’ performance on the test dataset following training.

A comparison chart that highlights each model’s ROUGE and BLEU scores provides a summary of the findings. On the CNN/Daily-Mail dataset, the model that performs the best for text summarization is the one with the highest scores. The code is available in^21^.

**Fig. 7:**
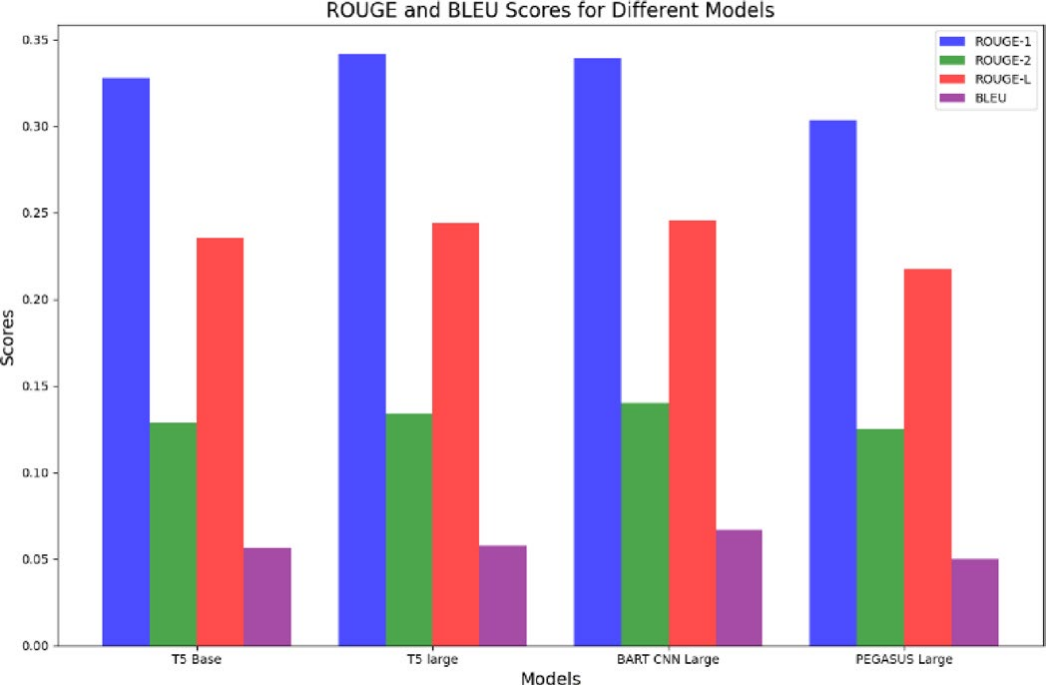
Combined bar chart.

The performance metrics after training for various methods is shown in Table 8. Also, the Fig. 7 shows a visual comparison of the ROUGE-1, ROUGE-2, ROUGE-L, and BLEU scores for the four deep learning models—T5-base, T5-large, BART CNN large, and PEGASUS-large, as shown by the combined grouped bar chart. It enables a clear and simple evaluation of each model’s performance across a variety of evaluation criteria by providing all metrics in a single graphic.

**Table 8:** Performance metrics after training.

Final performance (after training)					
Sl. no	Model	ROUGE-1	ROUGE-2	ROUGE-L	BLEU
1	T5-base	0.3279	0.1288	0.2354	0.0565
2	T5-large	0.3414	0.1340	0.2442	0.0575
3	BART CNN large	0.3388	0.1399	0.2458	0.0668
4	PEGASUS large	0.3037	0.1250	0.2173	0.0501

BART CNN-large is the best model for text summarization on the CNN/DailyMail dataset, regularly outperforming the other models in most measures, especially ROUGE-2, ROUGE-L, and BLEU scores. T5-large likewise performs well, particularly when it comes to ROUGE-1 scores. Although PEGASUS-large performs well in ROUGE-1, it falls short in other measures, indicating that it could use more adjustment and improvement. While it’s not the best, T5-base offers a reliable starting point for analysis.

These insights give a clear picture of the advantages and disadvantages of each model, directing future developments and optimisations for text summarising jobs.

**Fig. 8:**
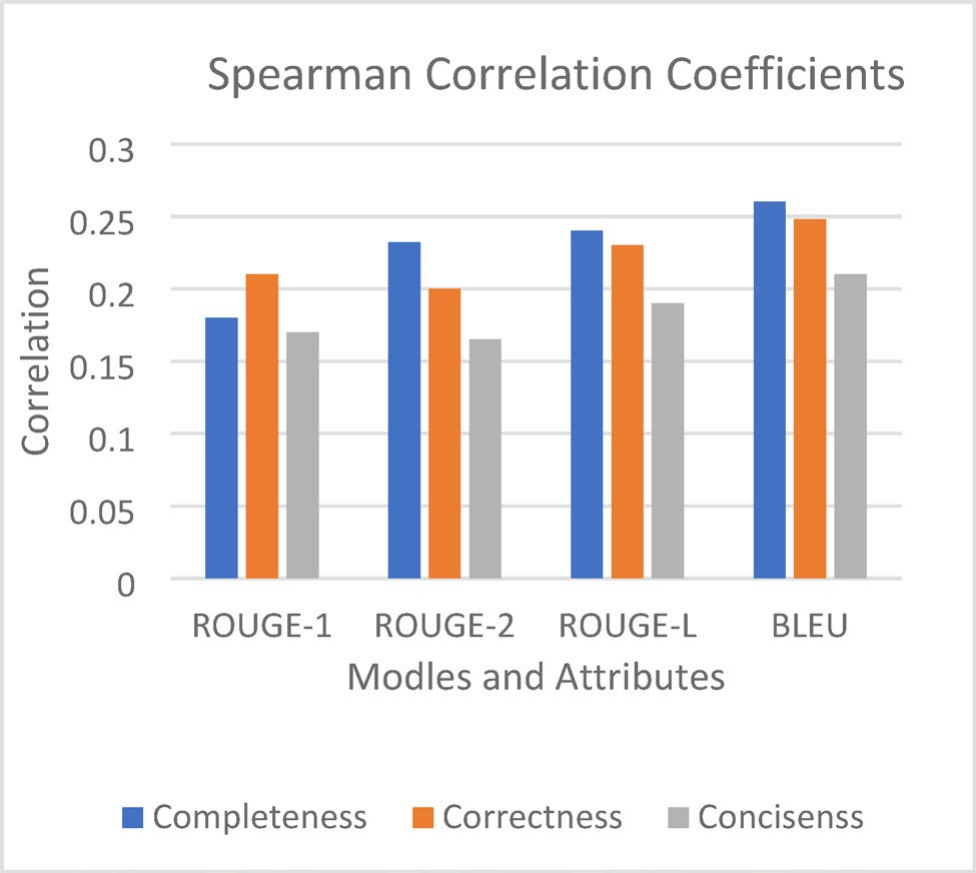
Correlation between NLP models and attributes.

For our further result analysis, we consider three major attributes completeness, correctness and conciseness. These attributes reflect the magnitude of the human assessments. Figure 8 given the Spearman correlations between the models and attributes. The correlation parameters indicates that BLEU perform significantly in terms of human assessments.

The metric METEOR is good in handling paraphrasing and recall, unlike BLEU. It is one of the good abstractive summarization metrics. The metric BLEU is for the translation and ROUGE for the summarization, whereas METEOR will take both translation and summarization. The advantages of the METEOR is it combines precision (P) and recall scores (R). The METEOR score is Score = Fmean × (1 − Penalty), where the $Fmean = \frac{10 \times P \times R}{{R + 9P}}.$ The METEOR scores for baseline models are shown in Table 9.

**Table 9:** The METEOR scores for baseline models.

Model	METEOR
Lead-3	18–20
Pointer-generator	22–25
BART-large	27–30
T5-base	25–28
PEGASUS	29–32

Now, we use METEOR to evaluate the summaries of the data set. The Table 10 shows the score value for different metric with 50 samples. The METEOR gives the score 0.27 where us ROUGE-1 gives the score 0.44. We show the score values for different metrics in Table 9.

**Table 10:** Scores for different metric.

Metric	Score
METEOR	0.27
ROUGE-1	0.44
ROUGE-2	0.21
ROUGE-L	0.41
BLEU	0.19

Finaly, we apply the paired *t* test to test the any significant difference in the mean values of the two models ROUGE and BLEU. The results of the paired *t* test indicated that there is a nonsignificant medium difference between ROUGE (mean is 32.8 and standard deviation is 56.8) and BLEU (mean is 33.5 and standard deviation is 57.3) with *t* value one and the *p* value is 0.361. Hence, the null hypothesis of our paired *t*-test indicate that the mean of the differences in the matched pairs is equal to 0. Further, we want to analyse the two models to check any significant difference on the evaluation metric. For that, we apply the Wilcoxon signed-rank test. This test shows that ROUGE is significantly better than BLEU.

## Conclusions

An extensive analysis of the use of deep learning models for automated text summarization is presented in this paper. Primarily, it focuses on assessing the CNN-DailyMail dataset, a benchmark in the field of NLP, to assess the performance of four deep learning models: T5-base, T5-large, BART CNN-large, and PEGASUS-large. The quality of the summaries produced by these models are evaluated using evaluation metrics such as rouge and bleu scores. The study emphasises how crucial text summaries are for controlling information overload, making the most use of available resources, and improving information accessibility.

The findings indicate that the BART CNN model outperforms the other models in terms of ROUGE and BLEU scores, suggesting it is the most effective for generating high-quality summaries from the dataset. This model’s superior performance can be attributed to its sophisticated architecture and extensive pre-training, which enable it to produce coherent and contextually accurate summaries.

The paper contributes to the advancement of text summarization techniques and provides valuable insights into the comparative performance of various deep learning models. Also, the methodological approach, which includes data cleaning, preprocessing, and rigorous model training and validation, ensures the reliability of the results.

In conclusion, the study demonstrates the potential of deep learning models, particularly the BART CNN model, in automating the summarization of textual content. The research underscores the ongoing efforts in the field of NLP to develop more efficient and accurate text summarization methods that can handle the complexities of natural language and provide precise, contextually aware summaries. The limitations of these studies only on the specific data set and model. This model can be generalised to any data set.

As a future study, Frequency-Inverse Document Frequency (TF-IDF) algorithm can be used for the text summarization and the results can be compared with other models. Medical reports summation is one of the important problems in the medical reporting. The proposed text summarization technique can be used for medical reports. Also, we need to contribute significantly to the fields of NLP and fully AI automated summarization.

## Data Availability

The datasets generated and/or analysed during the current study are available in the Kaggle repository, https://www.kaggle.com/datasets/gowrishankarp/newspaper-text-summarization-cnn-dailymail.

## Code availability

The code is available https://zenodo.org/records/17104346. The DOI No: 10.5281/zenodo.17104346.
